# Successful enrichment and recovery of whole mitochondrial genomes from ancient human dental calculus

**DOI:** 10.1002/ajpa.22960

**Published:** 2016-03-16

**Authors:** Andrew T. Ozga, Maria A. Nieves‐Colón, Tanvi P. Honap, Krithivasan Sankaranarayanan, Courtney A. Hofman, George R. Milner, Cecil M. Lewis, Anne C. Stone, Christina Warinner

**Affiliations:** ^1^ Department of AnthropologyUniversity of OklahomaNormanOK73019; ^2^ School of Human Evolution and Social ChangeArizona State UniversityTempeAZ85287; ^3^ School of Life SciencesArizona State UniversityTempeAZ85287; ^4^ Department of AnthropologyPennsylvania State University, University ParkPA16802; ^5^ Center for Bioarchaeological Research, Arizona State UniversityTempeAZ85287; ^6^ Institute of Human Origins, Arizona State UniversityTempeAZ85287; ^7^ Institute of Evolutionary Medicine, University of Zurich8057 ZurichSwitzerland

**Keywords:** ancient DNA, dental calculus, mitogenome, mitochondrial genome, next‐generation sequencing, in‐solution capture enrichment, NAGPRA, ethics, Mississippian culture

## Abstract

**Objectives:**

Archaeological dental calculus is a rich source of host‐associated biomolecules. Importantly, however, dental calculus is more accurately described as a calcified microbial biofilm than a host tissue. As such, concerns regarding destructive analysis of human remains may not apply as strongly to dental calculus, opening the possibility of obtaining human health and ancestry information from dental calculus in cases where destructive analysis of conventional skeletal remains is not permitted. Here we investigate the preservation of human mitochondrial DNA (mtDNA) in archaeological dental calculus and its potential for full mitochondrial genome (mitogenome) reconstruction in maternal lineage ancestry analysis.

**Materials and Methods:**

Extracted DNA from six individuals at the 700‐year‐old Norris Farms #36 cemetery in Illinois was enriched for mtDNA using in‐solution capture techniques, followed by Illumina high‐throughput sequencing.

**Results:**

Full mitogenomes (7–34×) were successfully reconstructed from dental calculus for all six individuals, including three individuals who had previously tested negative for DNA preservation in bone using conventional PCR techniques. Mitochondrial haplogroup assignments were consistent with previously published findings, and additional comparative analysis of paired dental calculus and dentine from two individuals yielded equivalent haplotype results. All dental calculus samples exhibited damage patterns consistent with ancient DNA, and mitochondrial sequences were estimated to be 92–100% endogenous. DNA polymerase choice was found to impact error rates in downstream sequence analysis, but these effects can be mitigated by greater sequencing depth.

**Discussion:**

Dental calculus is a viable alternative source of human DNA that can be used to reconstruct full mitogenomes from archaeological remains. Am J Phys Anthropol 160:220–228, 2016. © 2016 The Authors American Journal of Physical Anthropology Published by Wiley Periodicals, Inc.

Ancient DNA (aDNA) studies have long focused on analyses of bone and dentine. However, there are many instances in which these tissues are unavailable for study, either because permission for destructive analysis of skeletal remains cannot be obtained or because the skeletal remains that are available are insufficiently preserved. Moreover, while many descendent communities have generally favorable attitudes toward scientific analysis of human remains, others have raised cultural and ethical concerns, especially regarding destructive analyses (Kaestle and Horsburgh, 2002; Mayes, 2010). Specific objections to the scientific analysis of human remains vary greatly across cultures, and confusion arises because neither “destructive analyses” (Katzenberg, 2001) nor “human remains” are well defined (McManamon, 2000).

In North America, laws such as the Native American Graves Protection and Repatriation Act (NAGPRA) have given federally recognized Native American tribes, Alaska Native villages, and Native Hawaiian organizations the legal right to claim and manage their ancestral human remains, including repatriation and potential scientific investigation (McManamon, 2000). Destructive analysis of skeletal remains is a highly sensitive topic for many repatriation committees and institutions holding skeletal remains (Dongoske, 1996; Killion and Molloy, 1999; Kosslak, 1999; O'Rourke et al., 2005; Cast et al., 2010), and thus there is a great need for identifying alternative sources and methods for obtaining ancient DNA that do not involve the destruction of skeletal material. While alternative sources of ancient DNA are eligible for repatriation, the fact that they are not part of the human anatomy may ease some concerns about proceeding with scientific collaborations that involve destructive analysis.

Nondestructive extraction methods that do not damage sample structural integrity have been developed for the recovery of mitochondrial and nuclear DNA from museum and archaeological samples (Rohland et al., 2004; Bolnick et al., 2012; Hofreiter, 2012). These methods focus on the extraction of DNA from the surfaces of bones, teeth, and skin. However, these methods can be problematic if DNA preservation is poor or if surface contamination is high. Alternatively, a number of other sources of ancient human DNA are known in the archaeological record, including coprolites (preserved feces) (Poinar et al., 2001; Gilbert et al., 2008b), quids (chewed and expectorated plant matter) (LeBlanc et al., 2007), and aprons (menstruation garments) (LeBlanc et al., 2007). However, in most cases such sources are rare, being preserved only under exceptional circumstances, and many have culturally, geographically, or temporally limited distributions.

Recently, dental calculus has been identified as an abundant, nearly ubiquitous, and long‐term reservoir of ancient host‐associated biomolecules, including human DNA (Warinner et al., 2014b, 2015a). Dental calculus (tartar) is the product of *in vivo* calcification of dental plaque on the dentition. Calcification occurs when calcium phosphate ions present in saliva and gingival crevicular fluid (GCF) precipitate within dental plaque deposits, forming sequential mineralized layers corresponding to discrete plaque calcification events through time (Schroeder, 1969; White, 1991). The biological content of dental calculus is primarily microbial in origin (Warinner et al., 2014b), and its parent material, dental plaque, is estimated to contain >200,000,000 microbial cells per milligram (Socransky and Haffajee, 2005). In addition to microbes (bacteria and archaea), dental calculus also contains trace amounts of dietary (plant and animal) biomolecules and microfossils, viral DNA (primarily bacteriophages, or bacteria‐infecting viruses), and human DNA and proteins (Warinner et al., 2014a, 2014b).

Human DNA was initially detected within archaeological dental calculus by targeted PCR amplification of mitochondrial DNA (mtDNA), followed by haplogroup inference using restriction enzyme digestion (Black et al., 2011) or conventional cloning and Sanger sequencing of the hypervariable region I (HVRI) (De La Fuente et al., 2013). The subsequent application of shotgun metagenomics using next‐generation sequencing (NGS) technology further confirmed the presence of both mitochondrial and nuclear DNA within archaeological dental calculus, and moreover provided an estimate of its overall relative abundance: approximately 0.5% of all identifiable DNA sequences in one deeply sequenced sample (Warinner et al., 2014b). Given that dental calculus is the richest known source of aDNA in the archaeological record, with reported DNA yields routinely exceeding 100 ng mg^−1^ in well‐preserved samples (Warinner et al., 2015a), dental calculus represents a potentially important source of ancient human DNA.

The origins of human biomolecules within dental calculus have been recently elucidated by proteomics. To date, more than 60 human proteins have been identified within dental calculus, including follicular dendritic cell‐secreted protein, alpha amylase I, and hemoglobin, suggesting that GCF, saliva, and blood, respectively, are the primary sources of human biomolecules in dental calculus (Warinner et al., 2014b). However, while dental calculus contains human DNA and proteins, it is not a human tissue. The cellular content of dental calculus is almost exclusively microbial, with few or no human cells present. Dental calculus is a microbial biofilm that acquires human DNA and proteins passively, primarily through host secretions and immunity‐associated processes, such as NETosis (Ryder, 2010; Brinkmann and Zychlinsky, 2012). Thus, dental calculus is a host‐associated microbial substrate. It is the same material that dental hygienists remove during routine dental cleanings, and it is not an integral or vital part of the human body.

Given the potential for dental calculus to serve as an alternative source of human DNA in cases where destructive analysis of skeletal material is not possible, we sought to test whether dental calculus could serve as a source of genomic‐scale information. In this study, we report the successful recovery of whole mitochondrial genomes, or mitogenomes, from prehistoric North American human dental calculus using in‐solution capture and enrichment techniques, followed by high‐throughput NGS. Additionally, we compare NGS library construction protocols using three different DNA polymerases to evaluate the benefits and disadvantages of using proofreading versus non‐proofreading high fidelity enzymes to reconstruct mitogenomes from low‐template starting material. Overall, we find that mitogenome reconstructions are improved by the use of proofreading enzymes and high coverage sequencing.

## MATERIALS AND METHODS

### Study population

**Figure 1:**
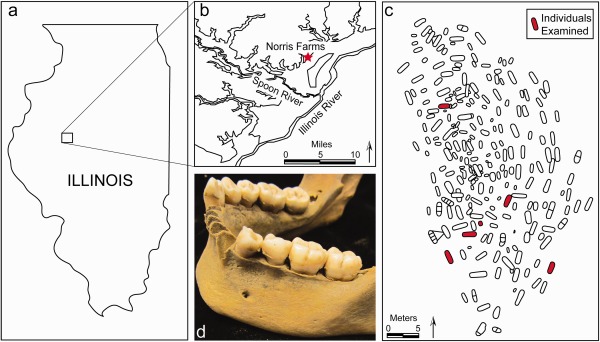
Norris Farms #36 cemetery and individuals sampled in this study. (**a**) Location of Norris Farms #36 cemetery within west central Illinois. (**b**) Enhanced view of the location of Norris Farms (star) with respect to the Illinois and Spoon Rivers. (**c**) Map of the cemetery with burial locations indicated. Individuals analyzed in this study are highlighted. (**d**) Close‐up view of dental calculus deposits on the mandibular dentition of NF‐95.

Dental calculus was analyzed from Norris Farms #36 (Fig. 1), a late prehistoric Oneota cemetery located in west‐central Illinois dating to about 700 BP (Milner and Smith 1990). This cemetery was excavated in the mid‐1980s by the Illinois State Museum and contained 264 well preserved Oneota skeletons that were likely interred over a relatively short period of time, perhaps as few as one or two generations (Milner and Smith, 1990; Santure et al., 1990). These people were village agriculturalists who buried their dead in a bluff‐edge cemetery that overlooked the Illinois River floodplain. The loess soil favored skeletal preservation, as is common in that part of the Midwest.

The Norris Farms #36 cemetery was selected for analysis because bone samples from this site had been previously investigated using ancient DNA techniques (Stone and Stoneking, 1993, 1998, 1999), and mtDNA haplogroup assignments and HVRI sequences are available for 108 and 52 individuals, respectively, making it the most intensively studied pre‐Contact North American site to date. The Norris Farms #36 skeletal remains are unaffiliated with existing Native American tribes, and permission for destructive analysis was granted by the Illinois State Museum.

Dental calculus samples from six individuals were selected for study: NF‐15, NF‐47, NF‐95, NF‐108, NF‐217, and NF‐262 (Table 1). Of these, ribs from three individuals (NF‐15, NF‐217, and NF‐262) were previously unsuccessful for mtDNA haplogroup analysis and HVRI sequencing using targeted PCR, while three other rib samples (NF‐47, NF‐95, and NF‐108) successfully produced results for mtDNA haplogroup markers, HVRI sequences, and amelogenin gene sex identification markers (Stone et al., 1996; Stone and Stoneking, 1998). Paired dentine samples were additionally analyzed from two of these six individuals (NF‐47 and NF‐217), one that had previously failed mtDNA haplogroup analysis (NF‐217) and one that had been successful (NF‐47). Two modern dental calculus (MOD‐1 and MOD‐2) samples were also analyzed to provide comparative data on the proportion of human DNA in non‐archaeological dental calculus. Sample MOD‐1 is from an individual with type 2 diabetes mellitus and active dental caries; MOD‐2 is from a healthy individual with no dental disease.

**Table 1:** Sample information and ancient DNA extraction yields

Sample ID	Sample size (mg)	Normalized DNA yield (ng mg^−1^)	Total DNA yield (ng)
*Dental calculus*			
NF‐15c	23.0	12.5	286.8
NF‐47c	16.0	37.5	571.2
NF‐95c	22.9	19.6	449.4
NF‐108c	21.3	69.0	1470.0
NF‐217c	25.0	30.0	750.0
NF‐262c	23.1	42.1	972.0
*Dentine*			
NF‐47d	97.4	0.1	6.8
NF‐217d	87.2	0.6	49.0
*Non‐template control*			
N	–	–	a

a DNA measurement fell below detection limit.

### DNA extraction

DNA was extracted from archaeological dental calculus in a dedicated ancient DNA facility at the University of Oklahoma Laboratories of Molecular Anthropology and Microbiome Research (LMAMR) in accordance with established contamination control precautions and workflows. A non‐template extraction control (negative control) was processed alongside the experimental samples during all analytical steps to monitor for possible contamination. DNA was extracted from six dental calculus (NF‐15c, NF‐47c, NF‐95c, NF‐108c, NF‐217c, NF‐262c) and two dentine (NF‐47d, NF‐217d) samples (Table 1). Prior to extraction, dental calculus samples (16–25 mg) were UV‐irradiated for 1 min on each side, and dentine samples (87–98 mg) were cleaned by mechanical abrasion of the tooth root surface using a Dremel rotary tool, followed by UV‐irradiation for 1 min on each side. To remove remaining surface debris and contaminants, dental calculus and dentine samples were agitated in a 0.5 M EDTA solution for 15 min and decanted. The dental calculus and dentine samples were then resuspended in 1 ml of 0.5 M EDTA solution and incubated overnight at room temperature. A 100 μl proteinase K solution (>600 mAU ml^−1^; Qiagen) was then added and incubated at 37°C for 8 h, followed by continued digestion under agitation at room temperature until decalcification was complete. For dentine samples, the digestion buffer solution was refreshed after 48 h and the two supernatants were combined for subsequent analyses. After digestion, DNA was extracted by a previously described phenol‐chloroform separation protocol and purified by silica adsorption (Qiagen MinElute PCR Purification kit) (Warinner et al., 2014b) using a modified protocol to increase binding buffer volume (Dabney et al., 2013a). DNA was eluted into 30 μl of EB buffer and 1 μl of each extract was quantified using a Qubit High Sensitivity dsDNA assay (Life Technologies) (Table 1). Modern dental calculus was extracted in a modern DNA laboratory using the same protocols except that the decontamination steps were not performed. The modern dental calculus samples were additionally sheared by sonication to a target length of ∼200 bp prior to library construction.

### Shotgun library construction

Shotgun Illumina libraries were constructed using previously developed protocols (Meyer and Kircher, 2010), with minor modifications. In brief, for each sample, 100 ng of DNA (or 30 μl of sample extract for low‐yield dentine and control samples) was constructed into indexed libraries using the NEBNext DNA Library Prep Master set for 454 (E6070; New England Biolabs) according to manufacturer instructions, except that SPRI bead purification was replaced with silica adsorption (Qiagen MinElute PCR Purification kit). Blunt end adapters (IS1/IS3 and IS2/IS3) were prepared following Meyer and Kircher (2010) and used for ligation at a concentration of 0.5 μM in a final volume of 50 µl. The Bst Polymerase fill‐in reaction was inactivated after incubation by heating to 80°C for 20 min and then freezing overnight. For each archaeological sample, three shotgun libraries were completed by PCR amplification using indexed primers and the following DNA polymerases: Platinum *Taq* High Fidelity Polymerase (Life Technologies), Phusion Hot Start II (Thermo Fischer), and KAPA HiFi Uracil+ (Kapa Biosystems). PCR reagent set‐up and cycling conditions are summarized in Supporting Information Table 1. All negative controls were amplified for 20 cycles. The two modern dental calculus samples were prepared as above in a modern DNA laboratory using the KAPA HiFi Uracil+ protocol with eight amplification cycles. All libraries were purified by silica adsorption (Qiagen MinElute PCR Purification kit) and quantified using a Bioanalyzer 2100 High Sensitivity DNA assay (Agilent).

### Mitochondrial DNA capture and sequencing

Mitochondrial DNA capture and enrichment was performed at the Arizona State University Molecular Anthropology Laboratory. Libraries created using different enzymes were captured separately. Prior to mitochondrial capture, 10 µl of each library was divided into 1‐µl aliquots and reamplified in ten parallel reactions using AccuPrime Pfx (Life Technologies) as follows: 1 µl AccuPrime Pfx DNA Polymerase, 10 µl 10× AccuPrime Pfx reaction mix, 3 µl IS5 primer, 3 µl of IS6 primer, 1 µl template, and 82 µl water. PCR conditions were as follows: initial denaturation at 95°C for 2 min, followed by 9–10 cycles of denaturation at 95°C for 15 s, annealing at 60°C for 30 s, and elongation at 68°C for 1 min, followed by a final elongation step at 68°C for 5 min. For each sample, the amplified library aliquots were combined and purified using a Qiagen MinElute PCR Purification kit with the following modification: the EB buffer was preheated to 65°C before use. The libraries were then quantified using a Bioanalyzer 2100 DNA 1000 assay (Agilent) and by quantitative PCR (qPCR) using the KAPA Library Quantification kit (Kapa Biosystems). The libraries were pooled in equimolar amounts across seven capture pools of 2 µg DNA each as previously described (Maricic et al., 2010). The libraries created using Platinum *Taq* HiFi were split into two capture pools—the two dentine libraries formed the first pool and the six calculus libraries and the negative control formed the second pool. The libraries created using KAPA HiFi Uracil+ and Phusion HS II were each split into two pools, with libraries of similar DNA concentration (ng/µl) being pooled together. Both KAPA HiFi Uracil+ and Phusion HS II negative controls were captured together in a separate pool.

DNA baits for the complete human mitochondrial genome were produced from modern DNA extracts of five individuals of diverse ancestry using previously described primers (Meyer et al., 2007) and following previously reported protocols (Maricic et al., 2010). Prior to capture, the baits were quantified using a Bioanalyzer 2100 DNA 1000 assay (Agilent). The libraries were target‐enriched for the complete mitochondrial genome according to previously reported protocols (Maricic et al., 2010) with the following modification: the volume of the library pool was increased to 70 µl, so as to avoid loss of DNA due to evaporation. The captured libraries were quantified by qPCR using the KAPA Library Quantification kit (Kapa Biosystems), amplified using AccuPrime Pfx following the conditions described above, and purified using a Qiagen MinElute PCR Purification kit as described above. Library insert sizes were assessed using a Bioanalyzer 2100 DNA 1000 assay (Agilent) and the libraries were quantified by qPCR using the KAPA Library Quantification kit (Kapa Biosystems). Each group of enriched samples was pooled in equimolar ratios of 10 nM each and sequenced across two Illumina MiSeq runs using v2 2 × 150 bp chemistry at the DNASU Sequencing Core at Arizona State University. Four archaeological and two modern pre‐capture DNA libraries were selected for shotgun sequencing to determine the starting mtDNA content of the pre‐captured libraries: NF‐47c, NF‐47d, NF‐217c, NF‐217d, MOD‐1, and MOD‐2. These samples were pooled in equimolar amounts with 11 other samples and sequenced using Illumina HiSeq v2 2 × 100 bp chemistry at the Yale Center for Genome Analysis (YCGA).

### Data analysis

Sequence reads were processed using a bioinformatics pipeline optimized for ancient DNA analysis. Post‐capture paired‐end reads were merged and adapters were trimmed with the program SeqPrep with a minimum overall overlap of 11 bp (https://github.com/jstjohn/SeqPrep). Reads < 30 bp in length were discarded. Read quality was assessed pre‐ and post‐merging with FastQC (http://www.bioinformatics.babraham.ac.uk/projects/fastqc /). Reads originating from four of the 18 dental calculus libraries were impacted by chimera formation during pooled post‐capture amplification (Kircher et al., 2012; Hawkins et al., 2015) and were excluded from further analysis (NF‐15c KAPA HiFi Uracil+, NF‐108c Phusion HS II, and NF‐47c Phusion HS II and KAPA HiFi Uracil+). The resulting analysis‐ready reads were mapped to the revised Cambridge Reference Sequence (rCRS) (Andrews et al., 1999) using the Burrows Wheeler Aligner (BWA aln) v. 0.7.5 (Li and Durbin, 2009) with default parameters except that seeding was disabled (−1 1000) and edit distance was increased (‐n 0.01) to improve mapping accuracy of ancient DNA reads, as recommended by (Schubert et al., 2012). Nuclear mitochondrial sequences (NumtS) were identified as any reads exhibiting superior alignment to the human nuclear genome, and removed. Read mapping was manually corrected at the loci 3,105–3,107 and 8,281–8,289 using Geneious R8 software (Biomatters), and the regions 300–320 and 510–520 were masked because they are known to be mutation hotspots and highly heteroplasmic in modern individuals (Diroma et al., 2014). Picard Tools v 1.137 (http://picard.sourceforge.net) was used to remove PCR duplicates and reads with a Phred quality score < Q30. Additionally, reads mapping to more than one location were also discarded. Pre‐capture paired‐end reads were analyzed as described above except that alignment was performed to the full human genome (Hg18, NCBI Build 36.1, March 2006) (Lander et al., 2001). The substitution error rate (incorrect/correct high quality aligned nucleotides) for each of the DNA polymerases used in this study was calculated by comparing the reads generated using each enzyme library to the consensus mtDNA genome for that enzyme library using the above BWA settings and Picard Tools v 1.137.

Ancient DNA damage patterns were assessed on unmerged reads using MapDamage 2.0 (Jonsson et al., 2013), and read quality scores were modified with the rescale option to account for post‐mortem damage. Contamination levels were estimated using contamMix (Fu et al., 2013). A set of 311 full‐length mitochondrial genomes from around the world was used as the potential contaminant source. SNP variants were called using SAMtools and bcftools using the rescaled BAM file (Li et al., 2009). MtDNA haplogroup assignment was performed on positions with a read depth > 1 (coverage filtered) using HaploGrep 2.0 (Kloss‐Brandstatter et al., 2011), followed by manual confirmation of haplogroup‐defining mutations provided in Phylotree mtDNA tree Build 16 (van Oven and Kayser, 2009). The HaploGrep formatted .hsd file of mitochondrial sequence variants is provided as Supporting Information File 1. HVRI sequences determined in this study were compared to previously published sequences (Stone and Stoneking, 1998).

## RESULTS AND DISCUSSION

### Total DNA yield from calculus and dentine

Total DNA yield from dental calculus was found to exceed that of dentine by an average of >25‐fold (Table 1), a finding consistent with previous studies (Warinner et al., 2015a). Comparison of calculus and dentine pairs for NF‐47 and NF‐217 likewise produced similar results, with calculus DNA yields exceeding that of dentine by 84‐fold and 15‐fold, respectively (Table 1).

### Proportion of human DNA in calculus and dentine

Although rich in total DNA, the proportion of human DNA in dental calculus is low and variable. The two modern dental calculus samples in this study, MOD‐1 and MOD‐2, contained 9.1 and 0.1% total human DNA, respectively, a difference of nearly two orders of magnitude (Table 2). The proportion of mtDNA in modern dental calculus was much lower; ∼0.002% for both samples (Table 2). Consequently, the ratio of mtDNA to total human is not constant in dental calculus and differs by more than an order magnitude. The reasons for this are unclear, but may be related to differences in the cell types contributing to the human DNA component of dental calculus. For example, neutrophils, an abundant blood leukocyte involved in host innate immune response to dental plaque and calculus formation (Warinner et al., 2014b), contain 10–15 times fewer mtDNA genome copies compared to peripheral blood mononuclear cells (PBMC) (Maianski et al., 2004). However, during immune response, neutrophils utilize mtDNA as a structural molecule to bind cytotoxic proteins into neutrophil extracellular traps (NETs), which are then released onto advancing plaque biofilms (Yousefi et al., 2009). Host immune response and the NET activity of neutrophils at the site of plaque formation may thus influence the ratio of mitochondrial to nuclear DNA present within individual dental calculus samples.

**Table 2:** Sequencing statistics for pre‐capture shotgun libraries

Sample ID	Analysis ready reads^a^		Total human reads^b^	Proportion human	Total mtDNA reads^c^	Proportion mtDNA	Human DNA length (bp)^d^
*Modern dental calculus*							
MOD‐1		5,618,173	5,856	0.1042%	129	0.0023%	n/a
MOD‐2		7,957,021	723,984	9.0987%	150	0.0019%	n/a
*Ancient dental calculus*							
NF‐47c		3,672,296	1,961	0.0534%	6	0.0002%	59
NF‐217c		3,682,330	4,541	0.1233%	0	<0.0001%	63
*Ancient dentine*							
NF‐47d		8,377,464	132,145	1.5774%	3,228	0.0385%	56
NF‐217d		5,105,533	7,491	0.1467%	14	0.0003%	61

a Reads after adapter trimming, chimera removal, removal of sequences <30 bp, quality (Q30) filtering, and read merging.

b Total analysis ready reads mapped to the human genome Hg18, NCBI Build 36.1, release date March 2006.

c Total analysis ready reads mapped to the rCRS.

d Median fragment length of total human reads.

With respect to archaeological dental calculus, the proportion of mitochondrial DNA was found to be lower in dental calculus compared to dentine, both prior to and after capture enrichment of DNA libraries for mtDNA (Tables 2 and 3). This is consistent with the fact that dental calculus is primarily a calcified microbial biofilm. Prior to enrichment, the proportion of human mtDNA in dental calculus was very low (NF‐47, <0.0001%; NF‐217, 0.0002%). The proportion of human mtDNA in dentine prior to enrichment was higher (NF‐47, 0.0385%; NF‐217, 0.0003%), but still low (Table 3). Because more than 98% of the total reads in the dentine dataset are nonhuman in origin, postmortem taphonomic processes appear to be an important contributing factor in the low mtDNA proportions observed in dentine, which during life should exclusively contain human DNA. After capture enrichment, the proportion of mtDNA in both dental calculus and dentine increased substantially by several orders of magnitude (Tables 2 and 3). On average, the relative proportion of human mtDNA in dereplicated post‐capture DNA libraries for dental calculus and dentine was 0.41 and 1.37%, respectively (Table 3).

**Table 3:** Sequencing statistics and sample quality metrics for mtDNA captured libraries

Sample ID	Total analysis ready reads^a^		Total mtDNA reads^b^	Unique analysis ready reads^c^	Unique mtDNA reads^d^	Proportion endogenous mtDNA^e^	mtDNA length (bp)^f^	Authentic^g^	mtDNA coverage^h^
*Dental calculus*									
NF‐15c		507,781	4,187	455,131	1,598	0.35%	61	95.3%	6.8
NF‐47c		327,874	6,761	274,971	1,524	0.55%	68	95.8%	7.1
NF‐95c		1,283,872	29,838	877,058	3,145	0.36%	62	91.5%	13.9
NF‐108c		1,999,869	44,572	1,220,367	3,594	0.29%	86	96.6%	21.4
NF‐217c		2,036,850	72,331	1,235,623	4,239	0.34%	72	96.5%	21.4
NF‐262c		1,752,516	57,484	1,107,221	6,220	0.56%	76	96.3%	33.8
*Dentine*									
NF‐47d		4,066,147	1,521,276	2,914,828	33,572	1.15%	158	100.0%	325.2
NF‐217d		3,575,314	666,742	1,867,276	29,615	1.59%	88	98.7%	169.7

a Combined results for all three post‐capture library sets. Reads after adapter trimming, chimera removal, removal of sequences < 30 bp, quality (Q30) filtering, and read merging.

b Total post‐capture analysis ready reads mapped to rCRS.

c Dereplicated post‐capture analysis ready reads. Duplicate reads were defined as reads with identical end‐to‐end alignments > 60 bp, and removed.

d Dereplicated post‐capture analysis ready reads uniquely mapped to rCRS. Duplicate reads were removed using PicardTools default settings.

e Proportion of unique mtDNA reads in post‐capture unique analysis ready data set.

f Median fragment length of post‐capture unique mtDNA reads.

g Estimated proportion of authentic endogenous human reads using contamMix (Fu et al., 2013).

h Mean depth of coverage.

### Authenticity of ancient human DNA

The authenticity of the human reads obtained from archaeological dental calculus and dentine was assessed by examining damage patterns typical of ancient DNA (Briggs et al., 2007; Ginolhac et al., 2011; Jonsson et al., 2013). Briefly, authentic ancient DNA is known to exhibit characteristic damage that includes short DNA fragment lengths, a high rate of cytosine deamination at the termini of DNA fragments, and an enrichment of purine bases at DNA breakage sites. Each of these patterns was evident in both our pre‐ and post‐capture data sets. Pre‐capture median DNA fragment lengths were short and of similar length in both calculus and dentine, ranging from 56 to 63 bp (Table 2). Post‐capture median DNA fragment lengths for the same samples were longer, and differences were observed between dental calculus and dentine (Table 3). Post‐capture median fragment lengths in dental calculus were ∼9 bp longer than prior to capture, while post‐capture median fragment lengths in dentine samples were 27–102 bp longer than prior to capture, a substantial increase. In‐solution capture enrichment is known to bias towards the recovery of longer DNA fragments (Dabney et al., 2013a), and the length differences in captured DNA observed between calculus and dentine is likely related to the fact that the dentine samples started with a higher endogenous content in the pre‐capture libraries.

Despite the relatively low sequencing depth, both pre‐ and post‐capture libraries exhibited elevated rates of 5′ cytosine deamination, but, interestingly, we observed an approximately 2‐fold lower mean frequency of cytosine deamination at position 1 in dental calculus samples compared to dentine (Supporting Information Fig. 1). This pattern suggests that hydrolytic damage may be lower in dental calculus than in dentine, but greater sequencing depth and a larger sample size are necessary to determine if this is indicative of a broader trend.

Cytosine deamination was also higher in post‐capture libraries than in pre‐capture libraries (Supporting Information Fig. 1). In paired samples, post‐capture libraries contained 14–15% more damage in dental calculus samples and 59–72% more damage in dentine samples. This pattern is also likely related to the fact that the pre‐‐capture dentine samples started with a higher endogenous content that included a subset of longer, but more heavily damaged, DNA fragments. Finally, a DNA breakage pattern of 5′ −1 enrichment of purine bases was observed for all ancient samples (Supporting Information Fig. 2). Taken together, these results are consistent with ancient DNA and support the authenticity of the human reads in our data sets.

To estimate the proportion of contaminant mitochondrial sequences in our data set, we implemented the Bayesian‐based tool ContamMix (Fu et al., 2013). This tool models observed reads as a mixture of endogenous and contaminant mitogenomes and uses a Markov Chain Monte Carlo algorithm to estimate the probability that a given read is from a contaminant source. The mitochondrial reads in our data set are estimated to have a high endogenous content (91.5–100.0%) (Table 3). These results further support the authenticity of the human mtDNA sequences in our data set.

### Substitution error rate

Because successful library construction is a critical step in NGS sequencing, we compared the performance of three high fidelity DNA polymerases with different properties: KAPA HiFi Uracil+, Platinum *Taq* HiFi, and Phusion HS II. KAPA HiFi Uracil+ is a family‐B polymerase that has been designed (through inactivation of its uracil‐binding pocket) to tolerate and amplify over deaminated cytosine residues (uracils), the most common miscoding lesion observed in ancient DNA (Dabney et al., 2013b). Platinum *Taq* HiFi is a mixture of predominantly Platinum *Taq*, a family‐A polymerase that is largely tolerant of uracils, and a smaller quantity of *Pyrococcus* GB‐D polymerase, a family‐B polymerase that is intolerant of uracils. Platinum *Taq* HiFi has also been shown to efficiently amplify over >90% of uracil sites (Heyn et al., 2010). Because of their relative uracil tolerance, these enzymes should be more efficient at amplifying ancient DNA, but they are also expected to result in a higher substitution error rate due to amplification of miscoding lesions. By contrast, Phusion HS II, a proofreading *Pfu*‐based family‐B polymerase with an intact uracil‐binding pocket, disproportionately stalls at deaminated cytosines, terminating amplification of damaged sequences. Consequently, this enzyme is expected to amplify fewer ancient DNA molecules, but the resulting sequences should have a lower substitution error rate. Previous studies have reported lower error rates and higher extension termination rates for *Pfu*‐based polymerases (such as Phusion) compared to *Taq*‐based and other uracil‐tolerant polymerases (Heyn et al., 2010; Ginolhac et al., 2011; Wales et al., 2014). We observed similar trends in this study. For dental calculus samples, Phusion HS II libraries were observed to have a nearly 2‐fold lower substitution error rate (1.0%) than the Platinum *Taq* HiFi (1.7%) or KAPA HiFi Uracil+ (1.8%) libraries. Substitution error rates were higher in dentine for all enzymes (Phusion HS II, 1.6%; Platinum *Taq* HiFi, 2.4%; KAPA HiFi Uracil+, 3.4%), a pattern consistent with the higher degree of cytosine deamination observed in these samples (Supporting Information Table 2).

### Mitogenome reconstruction and haplogroup determination

Medium coverage (7–34×) whole mitogenomes were successfully reconstructed from the dental calculus of all six profiled individuals (Table 3), and haplogroup assignments matched those for paired dentine samples in this study and matched bone samples previously analyzed by targeted PCR of the HVRI region (Stone and Stoneking, 1998) (Table 4). All haplogroup assignments were consistent with Native American maternal ancestry, as expected for this pre‐Columbian assemblage. Importantly, whole mitogenome reconstruction from dental calculus was successful for the three individuals (NF‐15, NF‐217, and NF‐262) that had previously failed to yield amplifiable mtDNA from bone (Stone and Stoneking, 1998). Of the two dentine samples that yielded full mitogenomes in this study, one (NF‐217) had previously failed mtDNA haplogroup analysis using traditional PCR, thereby highlighting the importance of newer ancient DNA extraction and next‐generation sequencing methods in successfully recovering genetic material from ancient specimens. Combined consensus mitogenome sequences for each individual are provided in Supporting Information File 2.

**Table 4:** Mitochondrial haplogroup assignment of Norris Farms samples

Sample number	Haplogroup assignment^a^	Haplogroup rank	Stone and Stoneking (1998)^b^
*Dental calculus*			
NF‐15c	A2f1	0.979	–
NF‐47c	D1	0.921	D
NF‐95c	B2	0.936	B
NF‐108c	A2f1	0.998	A
NF‐217c	A2+(64)+@153	0.904	–
NF‐262c	B2	0.921	–
*Dentine*			
NF‐47d	D1	0.961	D
NF‐217d	A2+(64)+@153	0.937	–

a Full mitogenome sequences generated in this study were assigned to haplogroup using HaploGrep 2.0 (Kloss‐Brandstatter et al., 2011).

b Previously published haplogroup assignments based on PCR‐amplified HVRI sequences (Stone and Stoneking, 1998).

In paired comparisons of dental calculus and dentine from the same individual (NF‐47 and NF‐217), dentine outperformed dental calculus in the total number of recovered unique human mtDNA reads (Table 3). Mitogenome coverage depth was 14‐fold higher for dentine samples; however, when normalized for sequencing effort (total analysis ready reads analyzed) the difference drops to only 4‐fold. Thus, at present, dentine presents a preferred tissue for mitogenome reconstruction, but in cases where it is not possible to analyze dentine, dental calculus is a suitable substitute that requires only slightly more sequencing effort to achieve equivalent results.

Finally, for one sample where data for both dentine and calculus and all three enzymes were available (NF‐217), we compared consensus sequences for each enzyme, each sample type (dentine, calculus), and a merged data set of all sequences (Supporting Information File 3). In general, consensus between each of these data sets was very high. Only 0.2% of sites (*n* = 29) were found to be variable across the NF‐217 mitogenome, and all but one were transitions. Manual inspection of these sites revealed that the variants were predominantly found in the Platinum *Taq* HiFi libraries, a pattern that appears to be driven by low sequencing depth. By contrast, KAPA HiFi Uracil+, which was sequenced at much higher depth, had fewer variable sites in the consensus sequences, despite exhibiting the highest error rate of the three enzymes. These findings highlight the value of read depth in overcoming damage‐driven substitutions in high‐throughput sequencing experiments.

## CONCLUSION

Dental calculus is an emerging archeological source of host‐associated biomolecules. It is unique among archaeological substrates in that it is abundant, nearly ubiquitous, preserves over long‐time periods, and contains both host genomic and acquired health/disease‐related information. In this study, we demonstrate that archaeological dental calculus is a viable and readily available alternative source of high quality, mitochondrial DNA sufficient for full mitogenome reconstruction. Despite the relatively low proportion of mitochondrial DNA in dental calculus, its substantially higher total DNA content means that, per milligram, dental calculus contains as much, if not more, mitochondrial DNA than dentine. Improvements in capture enrichment efficiency are necessary to take full advantage of this property, but even current methods are sufficient for full mitogenome reconstruction from small quantities (<20 mg) of dental calculus. The ability to conduct ancestry studies using dental calculus opens new possibilities in ancient DNA research, especially in contexts where destructive analysis of skeletal material is not possible. Moreover, because dental calculus is less porous and thus less susceptible to degradation by environmental microbes than dentine or bone, it offers an alternative source of ancient human DNA that may persist when other skeletal tissues fail to yield DNA. The application of advanced genomic sequencing techniques to dental calculus continues to reveal new and exciting opportunities for better understanding diverse aspects of the human past.

## Supporting information

Supporting Information File 1

Supporting Information File 2

Supporting Information File 3
