# Supplementary material for: Successful enrichment and recovery of whole mitochondrial genomes from ancient human dental calculus
Source: Am J Phys Anthropol. 2016 Mar 16;160(2):220–8. doi: 10.1002/ajpa.22960 (PMC4866892; doi:10.1002/ajpa.22960)
Supplement: Supplementary file 1 — Supporting Information File 1 [file AJPA-160-220-s001.pdf]

Supplementary Information

Successful enrichment and recovery of whole mitochondrial genomes from ancient human dental calculus

Andrew T. Ozga, Maria A. Nieves-Colón, Tanvi P. Honap, Krithivasan Sankaranarayanan, George Milner, Courtney Hofman, Cecil M. Lewis, Jr., Anne C. Stone, Christina Warinner

SI correspondence to:
Christina Warinner, twarinner@gmail.com

Supplementary Table 1. DNA library substitution error rates

	Sample	Tissue	Total Reads	HQ Aligned Reads[a]	HQ Aligned Bases[a]	Error Rate[bc]
Phusion HS II	NF-15c	Calculus	444	444	33,371	0.0115
	NF-95c	Calculus	819	819	61,220	0.0091
	NF-217c	Calculus	1,513	1,513	125,188	0.0081
	NF-262c	Calculus	1,693	1,693	148,294	0.0105
					Mean Calculus	***0.0098***
	NF-47d	Dentine	29,856	29,856	3,513,573	0.0159
	NF-217d	Dentine	3,880	3,880	277,582	0.0163
					Mean Dentine	***0.0161***
					Mean Total	***0.0119***
Platinum HiFi	NF-15c	Calculus	1,209	1,209	83,403	0.0169
	NF-47c	Calculus	1,516	1,516	119,238	0.0172
	NF-95c	Calculus	1,237	1,237	86,110	0.0153
	NF-108c	Calculus	632	632	58,178	0.0183
	NF-217c	Calculus	1,236	1,236	95,906	0.0162
	NF-262c	Calculus	1,022	1,022	82,354	0.0173
					Mean Calculus	**0.0169**
	NF-47d	Dentine	33,509	33,509	5,195,634	0.0263
	NF-217d	Dentine	1,731	1,731	105,385	0.0206
					Mean Dentine	**0.0235**
					Mean Total	**0.0185**
KAPA HiFi +U	NF-108c	Calculus	3,095	3,095	313,174	0.0216
	NF-217c	Calculus	2,135	2,135	184,578	0.0159
	NF-262c	Calculus	4,284	4,284	395,619	0.0205
	NF-95c	Calculus	1,452	1,452	107,412	0.0155
					Mean Calculus	**0.0184**
	NF-217d	Dentine	29,467	29,467	2,922,347	0.0337
					Mean Dentine	**0.0337**
					Mean Total	**0.0214**

Notes:

[a]HQ signifies high quality

[b]Geneious R8 software (Biomatters) was used to generate consensus FASTA sequences and for subsequent read mapping.

[c]Substitution error was calculated using Picard Tools v 1.137 (http://picard.sourceforge.net)

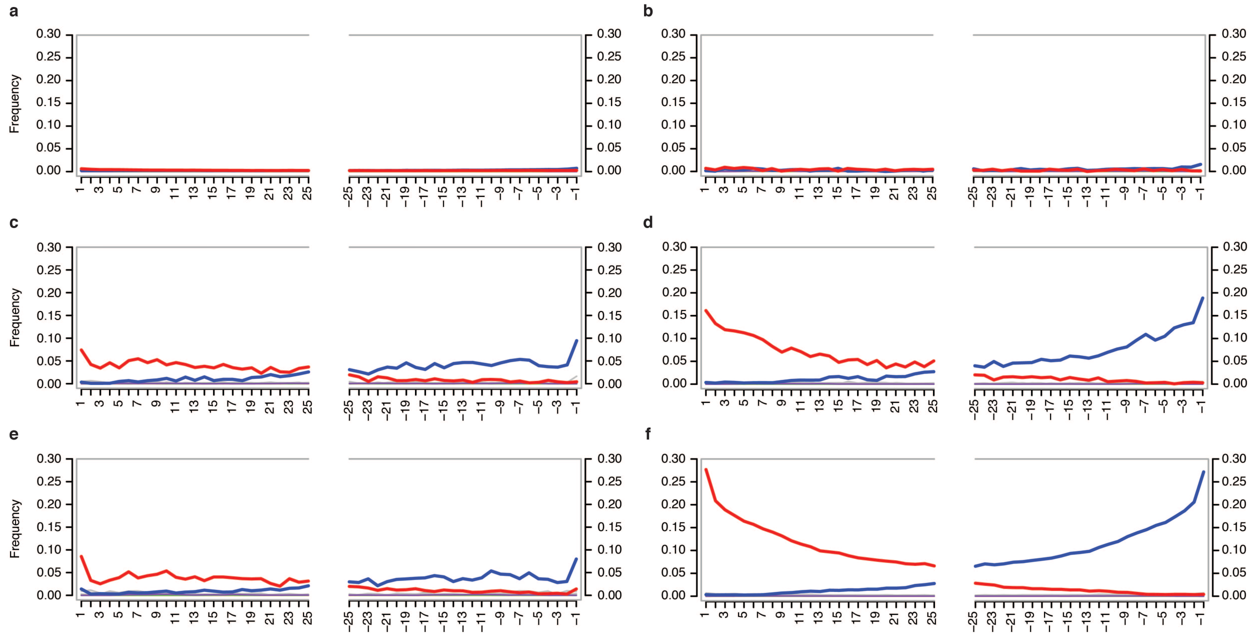

Supplementary Figure 1. **Damage profiles for one ancient and two modern samples.** (**a**) pre-capture MOD-1 dental calculus. (**b**) pre-capture MOD-2 dental calculus. (**c**) pre-capture NF-217 dental calculus. (**d**) pre-capture NF-217 dentine. (**e**) post-capture NF-217 calculus. (**f**) post-capture NF-217 dentine. C>T transitions are indicated in red; G>A transitions are indicated in blue. Modern samples (**a, b**) show little to no damage, while ancient samples (**c-f**) exhibit evidence of heightened miscoding lesions at the fragment termini. Note that damage levels are higher in dentine (**d, f**) than in calculus (**c, e**), and higher in post-capture libraries (**e, f**) than in pre-capture libraries (**c, d**). The remaining archaeological samples (NF-15, NF-47, NF-95, NF-108, NF-262) show similar damage patterns. Damage profiles were generated using MapDamage 2.0.

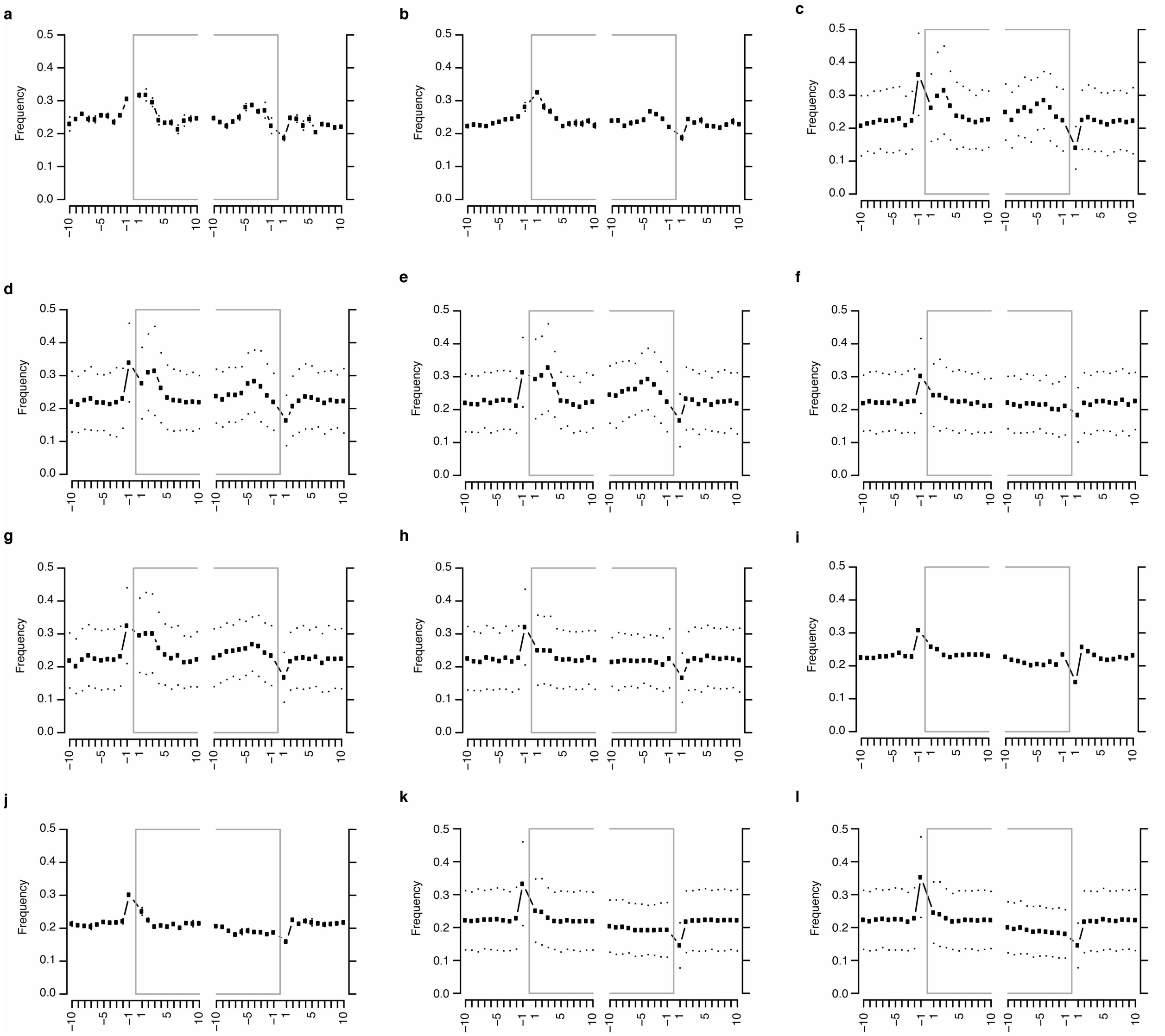

Supplementary Figure 2. DNA fragmentation profile for ancient dental calculus (a-h) and dentine (i-l) samples. A 5' -1 enrichment of the purine guanine is observed for all samples. (**a**) pre-capture NF-47c. (**b**) pre-capture NF-217c. (**c**) post-capture NF-15c. (**d**) post-capture NF-47c. (**e**) post-capture NF-95c. (**f**) post-capture NF-108c. (**g**) post-capture NF-217c. (**h**) post-capture NF-262c. (**i**) pre-capture NF-47d. (**j**) pre-capture NF217d. (**k**) post-capture NF-47d. (**l**) post-capture NF-217d. DNA fragmentation profiles were generated using MapDamage 2.0

Supplementary Data File 1. HaploGrep formatted input file (.hsd) of mitochondrial variants in Norris Farms samples.

Supplementary Data File 2. FASTA file of combined consensus mitogenome sequences for individuals NF-15, NF-47, NF-95, NF-108, NF-217, and NF-262.

Supplementary Data File 3. FASTA file of NF-217 mitogenome consensus sequences for dental calculus and dentine enzyme libraries.
